# Supplementary material for: A survey on awareness of the disease and pulmonary rehabilitation in bronchial asthma patients in the United Arab Emirates
Source: PLoS One. 2024 Jan 25;19(1):e0294463. doi: 10.1371/journal.pone.0294463 (PMC10810485; doi:10.1371/journal.pone.0294463)
Supplement: Attachment — Submitted filename: Reviewer 2 (2).docx [file pone.0294463.s002.docx]

Dear Dr. Sampath Amaravadi,

Plos One

Subject: Gratitude and Response to Your Insightful Feedback on Manuscript

Dear Editor,

We thank you and the reviewers for their valuable suggestions and comments. Kindly find below our responses and justifications to the comments given by the Reviewer 2

Reviewer #2,

I trust this message finds you well. Your encouraging words and shrewd insights into our study titled "A SURVEY ON AWARENESS OF THE DISEASE AND PULMONARY REHABILITATION IN BRONCHIAL ASTHMA PATIENTS IN THE UNITED ARAB EMIRATES" are deeply appreciated. I’m writing both to extend our heartfelt thanks and to address the critical points you’ve raised.

**Line Numbers 134 and 140:**

We sincerely apologize for the oversight and have amended lines 134 and 140 to include the missing author information, ensuring completeness and coherence.

**Table I Adjustment:**

Your suggestion to replace "statistics" with "Variables" in Table I is apt for clearer representation. This amendment has been duly made.

**Line Number 162 – Sentence Structure:**

The sentence structure discussing smoking history at line 162 was revisited and restructured for clarity, and we believe it now conveys the intended message more accurately.

**Data Duplication in Table 3 and Lines 166-168:**

We thank you for catching the partial data duplication. This has been rectified by eliminating redundancy while maintaining the integrity and clarity of the data presented.

**Limitations of the Study:**

We acknowledge the omission and have incorporated a section detailing the limitations of the current study, providing a balanced view and acknowledging the constraints encountered during our research.

**Conclusion and Its Consistency with Results and Discussion:**

You’ve pinpointed a crucial aspect, and in response, we've revisited our conclusion, results, and discussion sections to ensure consistency and coherent representation of aspects highlighted throughout. The conclusion now closely reflects the data and discussions presented in the preceding sections. The enlightening feedback you provided has significantly elevated our manuscript, and for that, we are sincerely grateful. All revisions have been highlighted in the attached manuscript for your convenience and easy reference. We ardently hope that the revisions made, in response to your astute observations, align with your expectations and bring our manuscript a step closer to being worthy of publication in [Journal Name]. Your time, expertise, and constructive critique have been indispensable in this journey, and we eagerly await further correspondence. Thank you once again for your invaluable input and looking forward to hearing from you soon.

Warm Regards,

Kalyana Reddy

Associate Professor, University of Sharjah
